# Reductions in substance use-related parenting behaviors after participation in a digitally delivered parenting intervention

**DOI:** 10.3389/fdgth.2026.1769417

**Published:** 2026-08-26

**Authors:** Amanda C. Trujillo, Samuel W. Rueter, Katherine A. Hails, Elizabeth A. Stormshak

**Affiliations:** 1 Department of Counseling Psychology and Human Services, College of Education, University of Oregon, Eugene, OR, United States; 2 Prevention Science Institute, University of Oregon, Eugene, OR, United States

**Keywords:** digital intervention, family prevention, harm reduction, parenting, substance use

## Abstract

The Family Check-Up Online (FCU-O) fills a unique gap in family prevention by providing digital, accessible content to support substance-involved parents outside of residential treatment settings to reduce the impact of substance use on parenting. We examined whether parents reported reductions in coping motivations for substance use (CM) and substance use-related parenting interference (PI) after completing the FCU-O substance use module. We further explored whether baseline substance use-related risk was associated with differential change in parenting outcomes over time.

**Method:**

Participants were parents (*N* = 180) of 1.5–5-year-old children assigned to the FCU-O within a randomized controlled trial and self-reported CM and PI via a pre- and post-assessment embedded within the app. We conducted linear mixed-effects models for each outcome.

**Results:**

Analyses revealed a significant effect of time for CM (*β* = −0.46, SE = 0.22, t = −2.13, *p* = 0.035), but not for PI (*β* = 0.05, SE = 0.18, t = 0.28, *p* = 0.783). Exploratory moderation analyses indicated that baseline substance use risk was significantly associated with higher levels of CM and PI at baseline, however, risk did not significantly moderate change over time for either CM or PI (*p* > 0.17 for each).

**Discussion:**

Findings suggest that parents endorsed less frequent CM after completing the FCU-O substance use module, while PI remained stable. These findings provide preliminary support for the FCU-O in improving substance-related coping behaviors and can guide future efforts to support substance-involved parents in parenting effectively and safely.

## 1 Introduction

The transition to parenthood is a significant risk factor for increased substance use (1). New parents experience role shifts and changes in family dynamics, which increase stress and prompt conflict and subsequent substance misuse. Nearly one in four U.S. children currently lives with a primary caregiver with a substance use disorder (2), and over 3 million children live in a household in which an adult misuses opioids (3). Opioid misuse among U.S. parents may be understood within a broader public health crisis, the opioid ‘epidemic’, that has overwhelmed healthcare and public health systems (4). Increases in nearly all forms of substance use among U.S. adults as a result of the COVID-19 pandemic (5), as well as a changing landscape of recreational cannabis legalization (6) and prescription opioid use (4), suggests prevalence of alcohol, cannabis, and opioid use among parents of young children.

Substance use may become maladaptive when it interferes with physical and psychological health (7–9), as well as the development and application of effective parenting practices. Further, the use of substances as a means of coping with distress is an established risk factor for mental health challenges, including depression, anxiety, and stress (10, 11). Unique stressors associated with parenting young children (e.g., navigating developmentally normative tantrums, sleep problems) necessitate increased research focus on parenting practices in relation to substance use. Research suggests that cannabis use is associated with negative parenting practices ranging from hostility and permissiveness (12) to physical abuse and corporal punishment, as well as neglect (13, 14). Though less robust, research on parenting and opioid use indicates similar potential for adverse parenting impacts, specifically, increased risk for physical abuse and neglect of children (15, 16). Overall, evidence points toward parental substance as a risk factor for adverse child development outcomes, including greater vulnerability for academic, social-emotional, and conduct problems, higher rates of illicit substance use, and increased involvement with justice and foster care systems (17).

Parenting challenges among parents who use substances (9, 18), likely caused by a combination of the physiological effects of substance use (8) and the underlying characteristics that contribute to substance use [e.g., emotion regulation challenges; (19)], necessitate preventive intervention for this population, particularly during the first three years of life when brain development is highly sensitive to environmental influences (20). Therefore, substance use that interferes with effective and responsive parenting during this critical period may be particularly consequential.

Stigma-related concerns about parenting in the context of substance use (21), as well as fears of child protective services involvement (22), limit disclosure and deter parents from seeking needed support. Even among those parents accessing substance use treatment, there is limited focus on parenting (23). Within a harm reduction framework, it is crucial to understand the evolving impacts of all levels of substance use on parenting behaviors and mental health outside of a narrow focus on abstinence. Urgency exists for wide-scale dissemination of parenting strategies that are easily accessible for families with limited access to support services, such as in rural areas (24), and those hesitant to disclose their substance use.

The Family Check-Up Online (FCU-O) is one such brief, five-module digital health intervention focused on supporting parenting skills (24) within a harm reduction framework. The FCU is an evidence-based program that improves both parenting skills (25) and parent mental health and wellbeing (26, 27) and reduces child behavioral and emotional problems (28–30). The online version includes self-directed modules accessed via a web-based application, paired with telehealth coaching. The early childhood version of the FCU-O was developed to support parents of young children with a history of substance use (24). Previous evaluations of the FCU-O, including the early childhood version (31), found it was associated with improvements in parent well-being, parenting skills, co-parenting, parenting self-efficacy, and reductions in child emotional problems (31, 32).

The current study examined substance use-related parenting impacts among parents of young children with histories of substance use or mental health challenges after completing the early childhood version of the FCU-O, which included a substance use content module. Specifically, we assessed whether participants assigned to the intervention evidenced decreased frequency of substance use to cope with stress or emotional distress and reduced substance use-related interference with parenting responsibilities. We also explored whether baseline variation in substance use risk was associated with differential change over time. Although prior research has not established whether parents with more complex substance use histories derive greater benefit from substance use-based parenting intervention, partly due to methodological limitations (33, 34), randomized controlled trials (RCTs) of the FCU and FCU-O have demonstrated that families experiencing greater contextual and psychosocial risk (i.e., child behavior problems, cumulative sociodemographic risk, and neighborhood deprivation) exhibit larger intervention effects than families with lower levels of baseline risk (28, 35–37). Given that the FCU-O was adapted for parents experiencing substance use and mental health challenges, we explored whether a similar pattern would emerge across levels of substance use risk. We hypothesized that 1) participants would report decreases in both coping-related substance use and substance-related parenting interference from baseline to follow-up, and 2) higher baseline substance use-related risk would be associated with (a) greater substance use-related parenting challenges at baseline, and (b) greater reductions in substance use-related parenting challenges after completing the intervention.

## 2 Method

### 2.1 Participants

**Figure 1:**
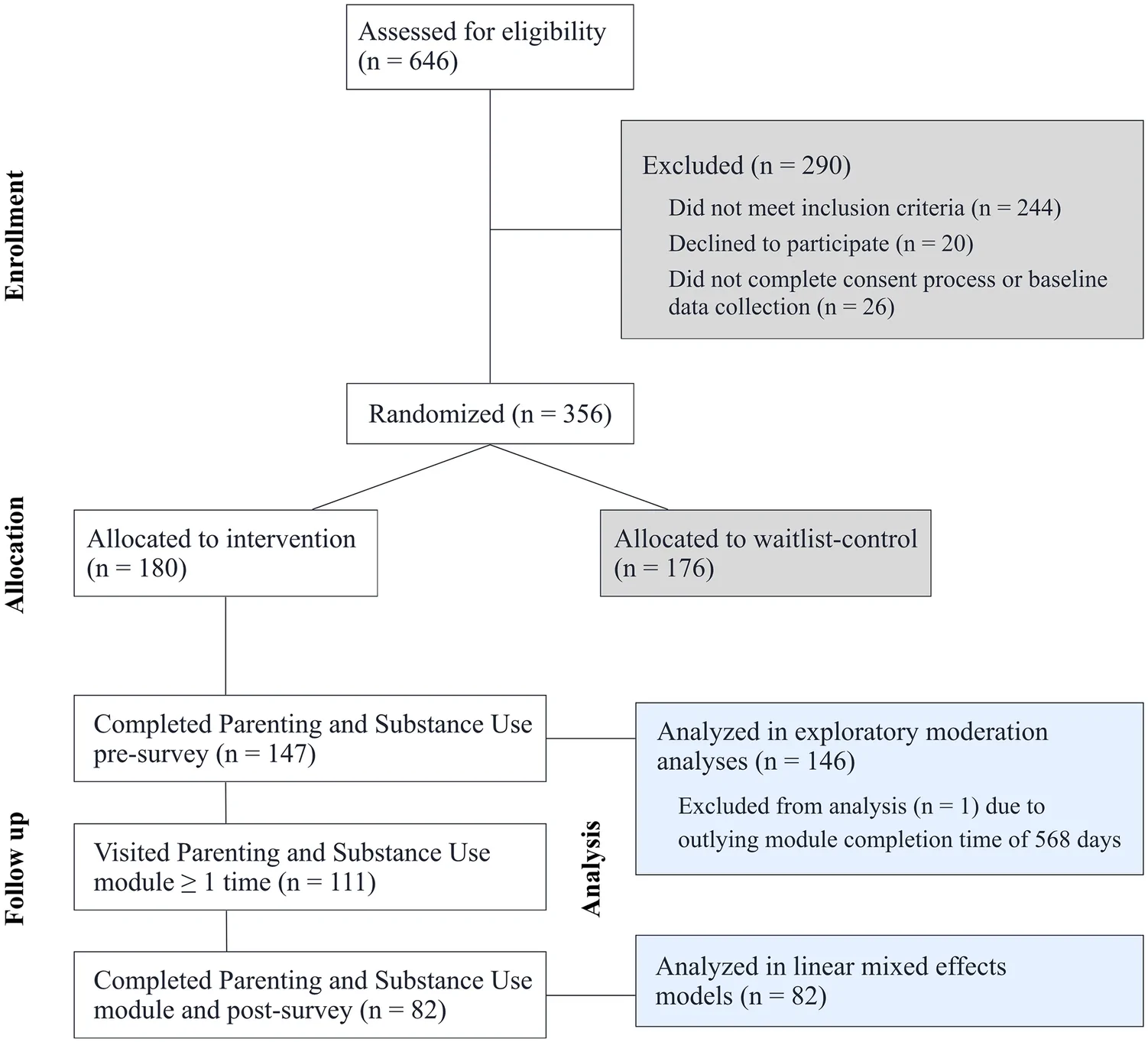
Participant recruitment and enrollment flowchart.

Participants were parents (*N* = 180) of 1.5–5 year old children living in the Pacific Northwest of the United States participating in the early childhood version of the FCU-O as part of a RCT. See Figure 1 for a participant flowchart and Hails et al. (31) for detailed study procedures, including recruitment, sampling, and adaptation procedures for the completed RCT. Participants were majority female (93.9%), and White, Non-Hispanic/Latinx (63.7%). Parents eligible for the RCT endorsed at least one indicator of risk for mental health challenges or substance misuse: past year binge drinking or recreational drug use (including cannabis), lifetime history of opioid misuse, or at least one depressive symptom, measured by an adapted version of the Patient Health Questionnaire-2 that assessed symptoms within the past year [PHQ-2; (38)]. Approximately one-third of the sample endorsed a history of opioid misuse, and another third endorsed clinically significant depression or anxiety symptoms.

### 2.2 Procedures

Study procedures were approved by the University of Oregon Institutional Review Board. Eligible parents lived with their child at least 50% of the time and had access to email and a smart phone with text messaging. Parents completed baseline questionnaires assessing family demographics, substance use history, and parenting and were then randomized to the intervention (FCU-O + telehealth parenting coach; *n* = 180) or waitlist-control condition (*n* = 176). The current sample represents all participants assigned to the intervention (*n* = 180).

### 2.3 FCU-O intervention protocol

Participants received access to the early childhood version of the FCU-O at baseline and were assigned a telehealth family coach. The FCU-O introduces parenting content across five modules, adapted from the Everyday Parenting Curriculum (39) and further modified for the FCU-O early childhood version to incorporate a focus on parent well-being and substance use. Each module contains content to support parenting skills, along with a brief self-assessment which provides automated feedback with strengths and areas for improvement. See Supplementary Material and Stormshak et al. (24) for detailed information on the FCU-O modules. Parents completed the five modules in order at their own pace and had access to the FCU-O for one year. On average, parents spent 87.84 min (*SD* = 54.0) in the app, and 73% of parents engaged with content from all five FCU-O modules. The telehealth coaching model was designed to include five to six 20–30-minute coaching sessions over three months. However, because it was intended to accommodate families’ specific needs and schedules, parents varied in the number of coaching sessions completed and the timing of app completion. Parents completed an average of 5.99 (*SD* = 4.7) coaching sessions, with an average session length of 29.44 min (*SD* = 15.5).

#### 2.3.1 Parenting and substance use module

The Parenting and Substance Use module in the early childhood FCU-O incorporates a focus on psychoeducation and harm reduction strategies for parents who use substances. Drawing on principles of motivational interviewing, parents are asked to consider their motivations for using substances and reflect on how substances impact their parenting. Next, parents view information about identifying triggers and managing cravings. The module also provides psychoeducation on substance use and how to maximize safety for children when substances are used in the home. Of the 180 participants assigned to the intervention, 82% (*n* = 147) completed the pre-survey for the Parenting and Substance Use module and 62% (*n* = 111) visited the module at least one time. Around half of participants (46%; *n* = 82) completed the module post-survey; these participants also completed all five FCU-O modules, including the Parenting and Substance Use module.

### 2.4 Measures

#### 2.4.1 Demographics and substance use history

Parents reported on demographic data at baseline, displayed in Table 1. Parents also reported on the number of visits made to therapy or counseling for alcohol or substance use-related concerns over the past 12 months, along with their current and historic use of alcohol, cannabis, prescription opioids, and heroin/fentanyl.

**Table 1:** Sample demographics.

Family Demographic Characteristics	Total Sample (*N* = 179)
PARENT'S AGE (YEARS)	M = 33.7 (SD = 5.6)
PARENT'S BIOLOGICAL SEX (Female)	93.9%
PARENT'S RACE/ETHNICITY	
White, Non-Hispanic/Latinx	63.7%
White, Hispanic/Latinx	11.2%
Multiracial, Non-Hispanic/Latinx	9.5%
Multiracial, Hispanic/Latinx	8.9%
American Indian/Alaska Native	1.1%
Asian	2.2%
Black/African American	0.6%
Native Hawaiian or other Pacific Islander	0.6%
Other/Unknown	1.7%
PARENT'S EDUCATION LEVEL	
Less than high school degree	9.0%
High school degree or GED	19.6%
Technical school, partial college, or associate's degree	38.0%
Bachelor's degree	22.3%
Graduate training or degree	10.1%
ANNUAL FAMILY INCOME	
< $15,000	20.7%
$15,000–$24,999	10.6%
$25,000–$34,999	12.3%
$35,000–$49,999	12.3%
$50,000–$74,999	16.8%
$75,000–$99,999	7.8%
> $100,000	16.2%
PARENT'S EMPLOYMENT STATUS	
Employed, part-time	21.2%
Employed, full-time	33.5%
Unemployed, looking for job	12.8%
Unemployed, not looking for job	31.7%

#### 2.4.2 Substance use app survey

Parents reported the frequency of coping motivations for substance use (CM) and substance use-related interference with parenting responsibilities (PI) via completion of a 9-item self-report assessment embedded within the “Parenting and Substance Use” module in the FCU-O app. Parents completed the measure at baseline upon first login to the app module and were prompted to complete the assessment again 6 weeks after registering for the app.

##### 2.4.2.1 Coping motives for substance use (CM)

Parents rated how often they are motivated to engage in substance use to alleviate feelings of depression, distract from a problem, and to increase self-confidence, on a scale from 0 (“Almost never/Never”) to 4 (“Almost always/Always”), with higher scores reflecting more frequent use of substances to cope. These 3 items, adapted from the Drinking Motives Questionnaire-Revised [DMQ-R; (40)], were selected as coping motivations for substance use in alignment with the Coping Motives subscale of the DMQ-R. Two additional substance use items included in the FCU-O app survey assessed enhancement motives (e.g., to increase positive affect) and craving reduction and were not included in the coping motives scale. Internal consistency for the 3-item scale was good (Cronbach's alpha = 0.85 for the pre- and 0.86 for the post-assessment).

##### 2.4.2.2 Substance use-related parenting responsibility interference (PI)

Parents indicated how often their use of substances over the past 3 months interfered with their ability to take care of, understand and connect with, pay attention to, or say kind things to their child, on a scale of 0 (“Never”) to 3 (“Almost Always”). Internal consistency for these 4 items, adapted from the National Survey on Drug Use and Health (41), was good (Cronbach's alpha = 0.88 for the pre- and 0.94 for the post-assessment).

##### 2.4.2.3 Substance use risk composite

To capture the heterogeneity in substance use histories within the sample, a continuous substance use risk composite was created using baseline indicators of substance use risk across alcohol, cannabis, prescription opioids, and heroin/fentanyl, including endorsement of lifetime repeated substance use (use at least once a month for 3 months in a row), substance use-related consequences (missing work, school, or other obligations or perceiving relational consequences due to use), past year substance use treatment, and lifetime exposure to frequent substance use within the home (e.g., “What was the most days per week that someone you lived with smoked marijuana?”). Items were combined such that higher scores reflected greater overall substance-related risk. The composite was included in exploratory moderation analyses to examine whether baseline substance use risk influenced changes in parenting-related substance use outcomes over time.

### 2.5 Data analysis

All data management procedures and statistical analyses were performed in RStudio (Version 2024.04.1 + 748).

#### 2.5.1 Within-group changes in parenting outcomes

To examine changes in parenting outcomes over time, linear mixed-effects models were conducted for each outcome variable. Assessment point [baseline (T1) vs. post-module follow-up (T2), with follow-up timing ranging from 14 to 224 days] was included as a fixed effect, and participant-level random intercepts were included to account for repeated observations nested within participants. Models adjusted for baseline demographic covariates, including parent age, gender, race, and education. Linear mixed-effects modeling allowed inclusion of all available data, including participants with incomplete follow-up data.

To evaluate potential attrition bias, baseline demographic, mental health, and substance use-related parenting outcomes were compared between participants who completed both baseline and follow-up assessments and those who completed baseline assessments only. Group differences were examined using multiple analyses of variance (MANOVAs) for continuous variables and chi-square tests for categorical variables.

Exploratory moderation analyses examined whether baseline substance use risk was associated with differential change over time by including a time-by-risk interaction term in each model. Additional sensitivity analyses examined whether module completion time was associated with outcomes among participants who completed follow-up assessments. One participant was excluded from analyses due to an outlying follow-up completion time of 568 days.

## 3 Results

### 3.1 Preliminary analyses: Demographics and missing data

Demographic characteristics are displayed for the total sample in Table 1. Descriptive statistics for the pre- and post- intervention CM and PI among participants who completed both pre- and post-surveys (*n* = 82) are reported in Table 2. Time spanning pre- and post-assessment ranged from 14 to 224 days (M = 72.7, SD = 38.6). Little's (42) Missing Completely at Random (MCAR) test was conducted to assess missingness in baseline demographic, mental health, and outcome variables. Results were non-significant [X^2^ (53) = 63.6, *p* = 0.152], suggesting no evidence of systematic missingness across observed variables. Baseline comparisons between participants who completed both pre- and post-assessments and those with partial or no follow-up data indicated no significant differences across demographic, mental health, or parenting variables. Consistent with study attrition, missing data were primarily concentrated across follow-up outcome variables. Linear mixed-effects models were used to retain all available data under the assumption of missing at random.

**Table 2:** Means and standard deviations on pre- and post-survey measures.

Outcome	Time	N	M (SD)	Change from T1 to T2
Coping motives for substance use (CM)	T1	146	2.71 (3.74)	–
	T2	81	2.09 (3.90)	−0.62
Parenting responsibility interference (PI)	T1	146	0.53 (1.55)	–
	T2	81	0.59 (1.56)	0.06

### 3.2 Within-group changes in parenting outcomes

**Figure 2:**
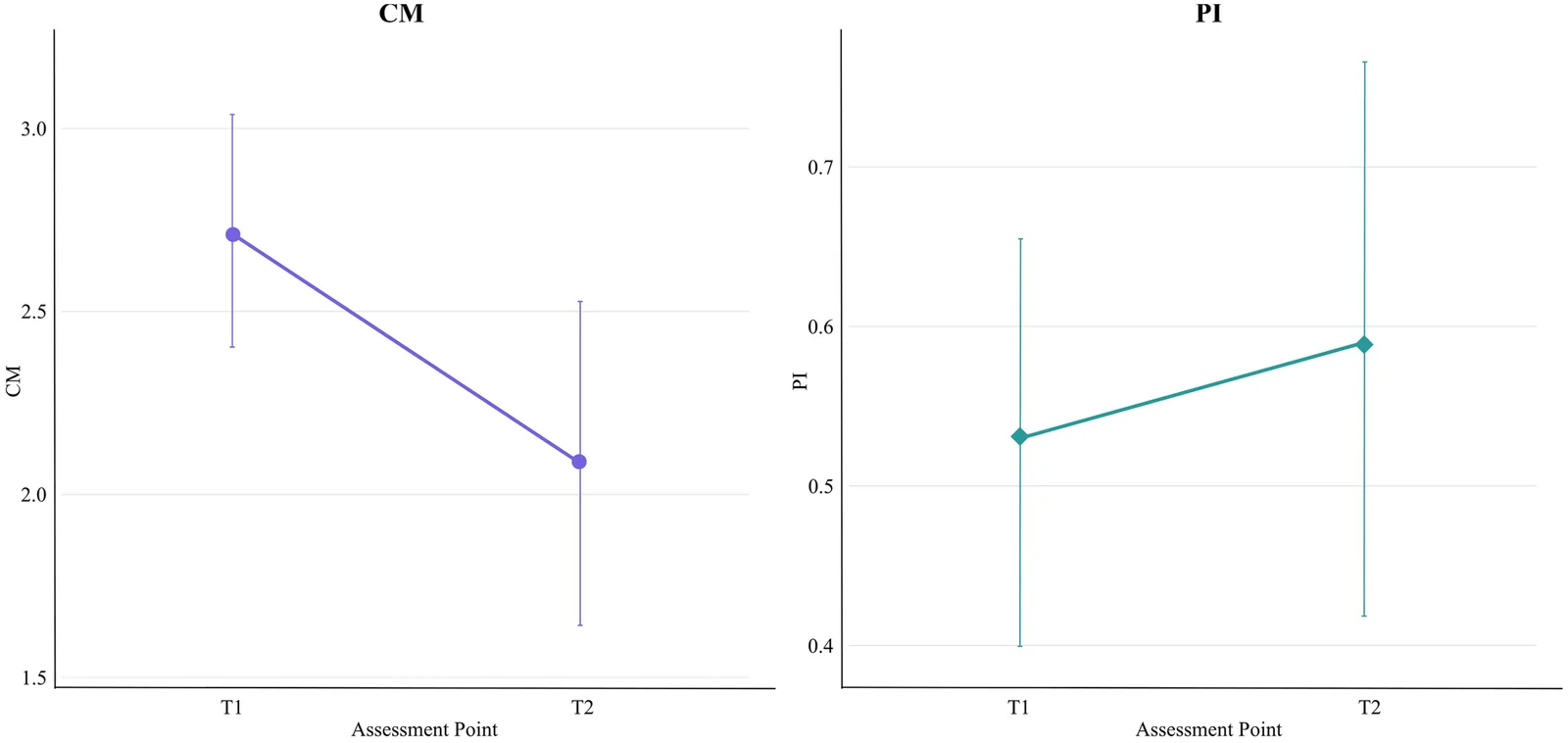
Estimated marginal means: Coping motives for substance use (CM) and parenting responsibility interference (PI).

Within-group changes in estimated marginal means for CM and PI are presented in Figure 2.

#### 3.2.1 Coping motives for substance use (CM)

Results indicated a significant and small decrease in CM from baseline to follow-up in the intervention sample, adjusting for covariates including parent age, gender, race, and education (*β* = −0.46, SE = 0.22, t = −2.13, *p* = 0.035). The effect remained statistically significant after adjusting for module completion time (*β* = −0.54, SE = 0.23, t = −2.39, *p* = 0.019).

#### 3.2.2 Parenting responsibility interference (PI)

No significant change in PI was observed from baseline to follow-up in the current sample (*β* = 0.05, SE = 0.18, t = 0.28, *p* = 0.783). Similarly, the effect of time remained nonsignificant after including module completion time (*β* = 0.07, SE = 0.20, t = 0.37, *p* = 0.710).

#### 3.2.3 Exploratory moderation by baseline substance use risk

Exploratory linear mixed-effects models examined whether baseline substance use risk moderated changes in outcomes over time. A significant and small to moderate main effect of risk was observed for both CM (*β* = 0.70, SE = 0.15, t = 4.63, *p* < 0.001) and PI (*β* = 0.37, SE = 0.10, t = 3.62, *p* < 0.001), indicating higher baseline levels of substance-related parenting challenges at baseline. However, the time × risk interaction was not significant for either outcome (*p* > 0.17 for each), suggesting that change over time did not differ as a function of baseline risk.

## 4 Discussion

The aim of this study was to examine whether parents assigned to the FCU-O experiencing substance use or mental health challenges reported reductions in substance use-related parenting impacts after completing the intervention. Namely, we examined whether parents reported decreases in CM and PI. We hypothesized that baseline level of substance use risk would 1) predict higher levels of CM and PI at baseline, and 2) moderate parenting changes, such that higher risk would predict greater reductions in CM and PI at follow-up.

### 4.1 Summary of findings

Results indicate that parents endorsed less CM after completing the FCU-O. No significant reductions in PI were observed among the total sample from baseline to follow-up. Parents who endorsed higher baseline levels of substance use risk did report higher levels of CM and PI at baseline, although risk levels did not impact changes in these outcomes over time.

These findings provide preliminary evidence that participation in the early childhood version of the FCU-O may support reductions in CM among parents experiencing substance use or mental health challenges. This pattern is consistent with broader research suggesting that parenting-focused and emotion regulation-based interventions may reduce reliance on substances as a coping strategy by increasing adaptive stress management skills and strengthening parental self-efficacy (43, 44). Reductions in substance use motivated by stress or emotional distress may be particularly relevant in parenting contexts, where parenting-related stress is a common trigger for substance use and emotional dysregulation among caregivers (1, 19, 45). Decreases in CM use may therefore reflect greater awareness of the relationship between substance use, emotional functioning, and parenting, as well as greater use of adaptive coping or parenting strategies introduced within the intervention, such as mindfulness practices or emotion regulation strategies.

In contrast, PI did not significantly change over time within this sample. PI may represent more chronic patterns that require longer intervention exposure, additional support, or broader environmental changes before measurable changes emerge. Ongoing contextual stressors (e.g., financial strain, housing instability, limited social support) may have contributed to substance use and PI in ways not captured in this study (46). Prior research has shown that changes in parenting behaviors and family functioning occur more gradually than changes in psychological coping or motivation (47), particularly among families experiencing significant contextual stressors (48, 49). Additionally, baseline levels of PI were relatively low across the sample, which may have limited our ability to detect meaningful change over time.

Consistent with hypotheses, parents with higher baseline levels of substance use risk reported greater CM and PI at baseline, suggesting the risk composite did capture meaningful variability in substance-related functioning within the sample. However, contrary to hypotheses, baseline risk did not moderate changes in outcome over time, meaning that although parents with greater substance-related risk entered the intervention with elevated difficulties, they did not demonstrate significantly different rates of improvement compared to lower-risk participants. One possible explanation is that the intervention targeted mechanisms such as stress management and emotion regulation that are relevant across levels of substance use risk. Alternatively, the relatively brief follow-up window may have limited the ability to detect differential trajectories of change across risk.

### 4.2 Strengths and limitations

In line with the harm reduction framework adopted by this study and the FCU-O curriculum, a key strength of this study is its focus on parenting-related impacts of substance use than frequency alone. Parents may understandably underreport substance use due to concerns about legal repercussions or child welfare involvement (50–52), and substance use frequency does not necessarily reflect functional impairment. Moreover, we adopt the approach that the harms of substance use are context-dependent and better captured through harms to personal functioning, and in this case, parenting impacts. Due to concerns about underreporting in this sample regarding substance use frequency, particularly for substances with greater stigma or potential legal consequences attached to misuse (e.g., heroin or fentanyl), this study is the first from this RCT to report on changes in substance use-related outcomes within this sample.

This strength should be considered alongside a few notable methodological limitations. Within the CM measure, although the three coping items were adapted from the validated DMQ-R (40) and demonstrated acceptable internal consistency in the current sample, the abbreviated three-item version used in this study has not been independently validated. Furthermore, without measuring substance use frequency, lower CM scores may reflect reduced coping-motivated substance use, reduced substance use frequency overall, or both (i.e., are parents continuing to use substances while motivated by other reasons, or are they using substances less?). The PI subscale more directly assesses substance use-related impacts on parenting, and changes in this subscale may be a more sensitive indicator of distal child development outcomes or reductions in parenting harms than the coping subscale alone.

Additional limitations include variability in follow-up timing and participant attrition. Although sensitivity analyses accounting for completion time produced comparable results and missing data analyses supported data being missing at random, the broad range in follow-up timing (14–268 days) may have limited detection of short-term intervention effects. A further limitation is the marked sex imbalance in the sample, with 94% of participants identifying as female. As a result, the present findings may be most reflective of maternal caregiving experiences, and, without the inclusion of fathers or caregivers of other gender identities in this sample, cannot be extended to a broader caregiver population. Although this pattern is consistent with parenting research, which overwhelmingly recruits and engages mothers (53, 54), it echoes the need for future research to examine samples diverse in caregiver gender.

The criteria used to capture baseline substance-related risk may pose a threat to ecological validity. Selection criteria included endorsement of lifetime repeated substance use, substance use-related consequences, past year substance use treatment, and lifetime exposure to frequent substance use within the home. These criteria do *not* differentiate between parents who are actively using substances, including alcohol, cannabis, and opioids, and those who may have used or misused substances many years ago. Additional dimensions of substance use risk, such as duration of use, polysubstance use, and perceived harm of current use, were not assessed in this study and could not be incorporated in the composite. Inclusion of these indicators may have provided a more comprehensive characterization of baseline substance use risk. Some participants who scored on the high end of risk may currently engage in substance use patterns better categorized as ‘low risk,’ and it is possible that participants struggling with the impacts of substance use on parenting (e.g., a parent who perceives dependence on alcohol or cannabis but has not received past year substance use treatment or noted clinically significant consequences of use) were not captured in this domain. Nevertheless, the elevated baseline rates of CM and PI among participants with higher baseline risk provide evidence that this group likely experienced more substance use challenges compared to lower risk.

Finally, as this study utilized a within-subjects design within a larger RCT, it should be noted that, without the ability to compare these findings to the control group, we are unable to differentiate between changes attributable to the intervention and simple effects of time. We emphasize the preliminary nature of these findings while highlighting their contribution to a limited but growing body of literature examining parenting interventions among substance-involved parents, a population that remains substantially understudied.

### 4.3 Implications

The findings from this study suggest that brief, digitally delivered parenting interventions incorporating substance use-specific content may support reductions in coping-motivated substance use among parents experiencing mental health or substance use challenges. These findings are especially important given the potential of the FCU-O to be disseminated in resource-limited settings where parents may not have access to or meet criteria for residential substance use treatment.

Parents with higher substance use-related risk at baseline reported greater coping-motivated substance use and parenting interference overall, suggesting substance use-specific content may still be relevant for parents experiencing substance-related challenges, even if brief effects emerge similarly across levels of risk. Future programs whose recruitment criteria span more than substance use concerns may benefit from identifying participants who may especially benefit from substance use content, which could increase relevance of the content and improve participant engagement.

It is, however, crucial to balance this consideration with the tendency for participants to underreport their substance use at baseline. Participants who may benefit the most from substance use treatment may be most likely to conceal their substance use, resulting in missed opportunities for treatment and discussion around safe substance use. Key considerations for future efforts to promote parent and family wellbeing in the context of substance use are: a) clearly define what constitutes substance use-related risks and harms (beyond reported frequency of use) with the aim of clearly assessing improvement in outcomes of interest, and b) balance participant desires for confidentiality with the information required from participants in order to deliver effective and tailored support.

The FCU-O fills a unique gap by providing digital, accessible harm reduction content to support parents outside of residential treatment to reduce the impact of substance use on their parenting. Although research on the original FCU model suggests sustained reductions in adolescent substance use (55–57), no research has examined whether parents completing the FCU-O report changes in the impacts of substance use on their parenting. These findings provide preliminary support for the FCU-O in improving parenting behaviors among parents who use substances and may inform future efforts to support parents in parenting effectively and safely.

## Data Availability

The raw data supporting the conclusions of this article will be made available by the authors, without undue reservation.

## Supplementary material

An appendix containing a) a detailed description of the five modules included in the FCU-O and b) measure items from the substance use in-app survey has been uploaded as Supplementary Material online.
