# Supplementary material for: Reductions in substance use-related parenting behaviors after participation in a digitally delivered parenting intervention
Source: Front Digit Health. 2026 Aug 26;8:1769417. doi: 10.3389/fdgth.2026.1769417 (PMC13553968; doi:10.3389/fdgth.2026.1769417)
Supplement: Supplementary file 1 [file Datasheet1.pdf]

Supplementary Material

1 Appendix

1.1 FCU-O Module Descriptions

1. Parent Wellness & Self-Care

FCU app content begins with the Parent Wellness & Self-Care module, which provides psychoeducation on the importance of parent mental health and promote self-care through skills that support child mental health and emotion regulation in the context of parenting. The module provides strategies for parents to engage in self-care, build their social support networks, establish healthy routines (e.g., exercise and sleep hygiene), and manage depression and stress through mindfulness, breathing, and scheduling enjoyable activities.

2. Parenting and Substance Use

The Parenting and Substance Use module was added specifically to the PYC version of the FCU Online to incorporate a focus on psychoeducation and harm reduction strategies for parents who use substances. Drawing on principles of motivational interviewing, caregivers are asked to explore the reasons they engage in substance use and to reflect on how substance use has impacted their parenting or relationship with their children. Next, caregivers view information about identifying triggers and managing cravings. The module also provides psychoeducation on substance use and how to maximize safety for children when substances are used (e.g., keeping substances in locked containers out of reach of children, only using substances when a second caregiver is present, encouraging relationships with people who do not promote substance use).

3. Positive Parenting

The Positive Parenting module educates parents on the importance of providing clear expectations and instructions for children, noticing and providing specific praise for desired behavior, and incorporating enjoyable time and play into family routines, with a focus on guiding parents through application of these skills to their own parenting. Finally, parents receive tips for relationship building through sensitive and responsive parenting and how incentives and rewards, as opposed to bribes, can effectively reinforce child behavior.

4. Proactive Parenting

Proactive Parenting underscores the importance of providing children with clear choices, breaking tasks into small steps, giving time warnings before transitions, and giving reasons for requests. Parents are encouraged to consider their child's capabilities and developmental norms to prepare for challenging situations and plan ways to prevent problem behavior. This module re-emphasizes the importance of establishing enjoyable time and child-led play into family routines.

5. Rules and Consequences

The Rules and Consequences module provides tools for establishing effective supervision and safety habits with children, as well as guidance for setting and enforcing rules. Parents are guided through

how to set enforceable limits and create logical consequences. This module also introduces ignoring unwanted behavior as a tool for reducing problem behavior.

For additional details regarding the development of the FCU-O modules and the adaptation from the in-person to online format, please see:

Stormshak EA, Matulis JM, Nash W, Cheng Y. The Family Check-Up Online: A telehealth model for delivery of parenting skills to high-risk families with opioid use histories. Front Psychol. 2021;12. doi:10.3389/fpsyg.2021.695967

1.2 Parenting and Substance Use Module Pre- and Post-Survey.

<u>Parenting Responsibilities Subscale.</u>

Please respond to the following questions by rating how often each occurred over the past three months (0 = Never, to 3 = Almost Always).

During the last three months, did drinking alcohol or using other drugs cause you to:

1. Have trouble taking care of your child?
2. Find it difficult to connect with and understand your child?
3. Say unkind things to your child?
4. Be unable to pay attention to your child?

<u>Coping Motives for Substance Use Subscale.</u>

Please respond to the following questions by rating how often each statement occurs (0 = Almost Never/Never, to 4 = Almost Always/Always).

How often do you drink or use drugs:

1. Because it helps you when you feel depressed or nervous?
2. Because you feel more self-confident or sure of yourself?
3. To forget about your problem?
